# Enabling precision medicine in neonatology, an integrated repository for preterm birth research

**DOI:** 10.1038/sdata.2018.219

**Published:** 2018-11-06

**Authors:** Marina Sirota, Cristel G. Thomas, Rebecca Liu, Maya Zuhl, Payal Banerjee, Ronald J. Wong, Cecele C. Quaintance, Rita Leite, Jessica Chubiz, Rebecca Anderson, Joanne Chappell, Mara Kim, William Grobman, Ge Zhang, Antonis Rokas, Louis J Muglia, Carole Ober, Sarah K. England, George Macones, Deborah Driscoll, Samuel Parry, Gary M. Shaw, David K. Stevenson, Joe Leigh Simpson, Elizabeth Thomson, Atul J. Butte, March of Dimes Prematurity Research Centers

**Affiliations:** 1 Institute for Computational Health Sciences, University of California, San Francisco, CA 94158, USA; 2 Department of Pediatrics, University of California, San Francisco, CA 94158, USA; 3 Northrop Grumman Health Solutions, Rockville, MD 20850, USA; 4 March of Dimes, White Plains, NY 10605, USA; 5 Enterprise Science And Computing, Inc., Rockville, MD 20850, USA; 6 March of Dimes Prematurity Research Center at Stanford, Department of Pediatrics, Stanford University School of Medicine Stanford, CA 94305, USA; 7 Perelman School of Medicine at the University of Pennsylvania, Philadelphia, PA 19104, USA; 8 Department of Obstetrics and Gynecology, Washington University in St Louis, St. Louis, MO 63110, USA; 9 Department of Human Genetics, University of Chicago, Chicago, IL 60637, USA; 10 Cincinnati Children’s Hospital Medical Center and University of Cincinnati College of Medicine, Cincinnati, OH 45267, USA; 11 Department of Biological Sciences, Vanderbilt University, Nashville, TN 37235, USA; 12 Department of Biomedical Informatics, Vanderbilt University Medical Center, Nashville, TN 37212, USA; 13 Department of Obstetrics and Gynecology, Feinberg School of Medicine, Northwestern University, Chicago, IL 60637, USA

**Keywords:** Reproductive disorders, Data integration, Data publication and archiving, Neonatology

## Abstract

Preterm birth, or the delivery of an infant prior to 37 weeks of gestation, is a significant cause of infant morbidity and mortality. In the last decade, the advent and continued development of molecular profiling technologies has enabled researchers to generate vast amount of ‘omics’ data, which together with integrative computational approaches, can help refine the current knowledge about disease mechanisms, diagnostics, and therapeutics. Here we describe the March of Dimes’ Database for Preterm Birth Research (http://www.immport.org/resources/mod), a unique resource that contains a variety of ‘omics’ datasets related to preterm birth. The database is open publicly, and as of January 2018, links 13 molecular studies with data across tens of thousands of patients from 6 measurement modalities. The data in the repository are highly diverse and include genomic, transcriptomic, immunological, and microbiome data. Relevant datasets are augmented with additional molecular characterizations of almost 25,000 biological samples from public databases. We believe our data-sharing efforts will lead to enhanced research collaborations and coordination accelerating the overall pace of discovery in preterm birth research.

## Background & Summary

In the last decade, the advent and continued development of genotyping and next-generation sequencing technologies has enabled researchers to generate a vast amount of molecular data. The current and ever-growing availability of public ‘omics’ databases, including Gene Expression Omnibus^1,2^, Array Express^3^, dbGAP^4,5^, and numerous other repositories, along with computational tools to reveal molecular drivers of disease at a network level, present a unique and new opportunity to refine current knowledge about disease mechanisms, diagnostics, and therapeutics. In addition, novel technologies are allowing high-throughput measurements of the human genome, epigenome, transcriptome, and immunome at the population level^6^. There have been several disease- and phenotype-specific efforts to aggregate datasets and to make them available in the public domain, with exemplary strides by the cancer community including The Cancer Genome Atlas (TCGA)^7^, which captures ‘omics’ profiling of tens of thousands of cancer samples; Cancer Cell Line Encyclopedia (CCLE), which focuses on molecular profiling of cancer cell lines; the Library of Integrated Network-Based Cellular Signatures (LINCS)^8^, which focuses on transcriptomic profiling of small molecules and other perturbations in cancer lines; and many others. However, other fields are lagging behind in the data-sharing realm. To this end, we aim to enable open data-sharing efforts in the fields of obstetrics and gynecology. Here, we present our March of Dimes (MOD) Database for Preterm Birth Research (http://www.immport.org/resources/mod), a unique resource that captures a variety of ‘omics’ datasets related to preterm birth (PTB).

PTB, or the delivery of an infant prior to 37 weeks of gestation, is a significant cause of infant morbidity and mortality. Globally, approximately 11% of infants are born prematurely every year, totaling nearly 15 million births. Infants born preterm are at risk for a variety of adverse outcomes, such as respiratory illness, blindness, and cerebral palsy, with associated complications resulting in nearly one million deaths each year^9–11^. Despite many attempts for preterm birth prevention, there is still an acute problem with prevalence rising according to the World Health Organization. Spontaneous preterm birth, accounting for two thirds of all preterm births, is considered a complex phenotype with no single known cause^12^ or biological basis^13,14^. One mechanism that has been associated with spontaneous preterm birth is chorioamnionitis, a condition associated with microbial infection of the amniotic fluids. Other suggested causes of PTB are progesterone deficiency, cervical insufficiency, disruption of the immune tolerance of the mother towards the fetus and disruption of the vaginal microbial balance, causing an inflammatory process^15–19^. Because of the complexity of the phenotype, a comprehensive integrative approach is needed to better understand the etiology of preterm birth and inform new diagnostic and therapeutic strategies.

The March of Dimes is dedicated to not only helping affected infants and families; but also preventing PTB. Only modest progress has been made in identifying the underlying causes of PTB, so the MOD has made this a top research priority. To foster a new model of collaboration with the hope of leading to transformative discoveries, six MOD Prematurity Research Centers have been launched. The goals are to integrate scientists from individual disciplines and to form innovative collaborations that can accelerate research discoveries^20^. The first Center was launched in 2011 at Stanford University School of Medicine to study infection and inflammation, transcriptomics and lead bioinformatics effort in PTB research. The second center, the MOD Prematurity Research Center - Ohio Collaborative, was opened in 2013 with a focus on evolutionary biology and genetics in PTB as well as molecular development of pregnancy, progesterone signaling and racial disparities in PTB. In 2014, two more centers were launched. On November 10, 2014 the MOD Prematurity Research Center at Washington University was established in St. Louis to apply bioengineering to study cervical remodeling and uterine contractility, as well as determining if chronodisruption is a risk factor for preterm birth. On November 17, 2014 the MOD Prematurity Research Center at the University of Pennsylvania was opened to address questions of cervical remodeling, placental development and bioenergetics in the context of PTB. The fifth center, MOD Prematurity Research Center University of Chicago-Northwestern-Duke was launched in 2015 with a focus on what causes premature birth, and specifically address six interrelated transdisciplinary research themes around gene regulation. The most recent center was launched in February 2018 at the Imperial College London with a focus on how the body recognizes and interacts with bacteria and other microbes in the birth canal that may increase the risk of premature birth.

There have been several previous efforts to aggregate and integrate genomics data for PTB research^21^. dbPTB^22^ is a resource that collects genomic data across a large number of studies focusing on linking information from published literature with data from expression databases, linkage studies, and pathway analyses to identify biologically relevant genes for testing in an association study of genetic variants and PTB. However, the database is limited to disease-gene and pathway associations. GEneSTATION^23^ is a comprehensive database that integrates cross-species genomic, transcriptomic, and evolutionary data to advance the understanding of the genetic basis of gestation - and pregnancy-associated phenotypes and to accelerate the translation of discoveries from model organisms to humans. This resource specifically focuses on evolutionary and genomic data and is not designed for capturing other ‘omics’ technologies that cannot be directly mapped to human genes or genetic elements (e.g., microbiome data). In addition, dbPTB does not allow for bioinformatics re-analyses of the processed datasets they aggregate and only summarizes the data at the results level; whereas, GEneSTATION is designed for exploratory data analyses and not as a tool for large-scale re-analyses or meta-analyses of pregnancy ‘omics’ data in general. A comprehensive resource that can capture more diverse types of data and enable data re-use and re-analyses is needed.

The MOD Database for Preterm Birth Research aims to organize scientific and clinical research data across the six MOD-Funded Prematurity Research Centers with the goal of enhancing research collaborations and accelerating the overall pace of discoveries in this field. Data from the Centers includes a diverse set of processed ‘omics’ data and results files as well as data generation protocols to support re-analyses and meta-analyses of the datasets. As of January 2018, the database references 9 studies across over 350 patients and with individual level molecular data on nearly 8,000 samples from 6 measurement modalities. Four additional large-scale GWAS studies across tens of thousands of patients are also included, for which only summary-level data are available. The repository includes genomic, transcriptomic, immunonological, and microbiome data that are available freely to the scientific community. Having all the data aggregated as part of the same resource, substantially extends the value the data allowing researchers to integrate the information and ask novel research questions.

## Methods

The database development was undertaken in collaboration with Northrop Grumman (NG) – Health Solutions, partner of the National Institute of Allergy and Infectious Diseases (NIAID) Division of Allergy, Immunology, and Transplantation (DAIT). Since 2004, NG has been the prime contractor for the ImmPort^24^ database and data-sharing portal, working with researchers at UCSF and Stanford to ensure that NIAID-funded discoveries serve as the foundation of future research. In order to host the MOD Database for Preterm Birth Research, we used the existing infrastructure, data model, and repository schema, known as ImmPort (www.immport.org)^24,25^. ImmPort encompasses immunological data across a wide range of diseases and conditions including several pregnancy-related studies. Study data includes over 50 diverse types of data including arrays, mass cytometry (CyTOF), enzyme-linked immunosorbent assays (ELISA), flow cytometry, and gene expression as well as others.

Datasets archived in ImmPort cover a broad spectrum of study-specific data including protocol designs, assay protocols, treatment and sampling time points, and subject demographics. Datasets are curated and organized by the ImmPort team prior to sharing with the scientific public. Curation efforts include adoption of community standards and controlled vocabularies across a broad spectrum of variables to improve data consistency within and across studies facilitating data re-use. Data upload templates instantiate standards and ontologies and are the result of ongoing outreach with domain experts, standards working groups, and research consortia. Data are de-identified. ImmPort has adopted best-practices in human study participant de-identification such that Health Insurance Portability and Accountability Act of 1996 (HIPAA)-restricted data are not captured in ImmPort. In addition, ImmPort references genetics data stored within the National Center for Biotechnology Information (NCBI) Database of Genotypes and Phenotypes (dbGaP), ensuring ImmPort data safeguards potentially identifying information.

**Figure 1:**
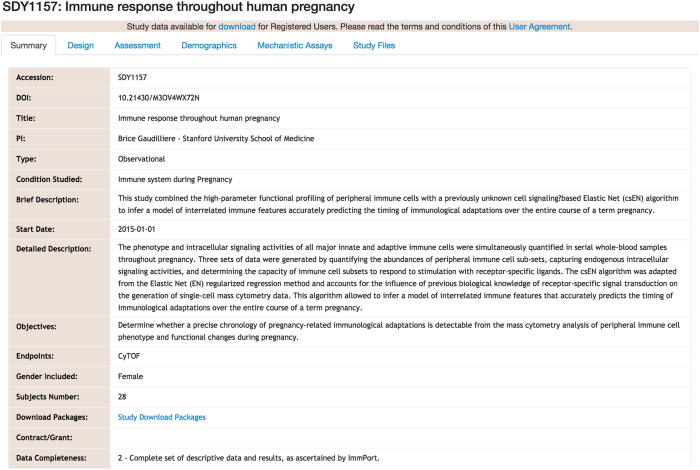
Screenshot of an example study indexed in the resource (SDY1157).

For each study (Fig. 1), a summary page is created, which contains the description, principal investigators (PIs), links to the publication, and external repositories with raw data. Only processed ‘omics’ data are stored in the repository to avoid duplication of effort. The information is drawn from the publication when available and confirmed by the PIs. Study design, types of assessments, and mechanistic assays with links to the protocols as well as the processed files are listed as separate tabs for each study. Finally, demographics are aggregated across all the samples for each study cohort where individual level data are available. Each subject is given a unique ID and when data from multiple assays is available for the same subject, the data can be accessed by the subject ID. All the human subjects work has been approved by the relevant IRB boards at each institution.

## Data Records

**Figure 2:**
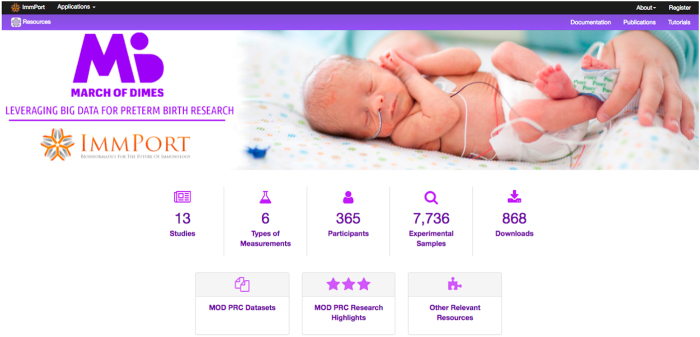
The MOD Database for Preterm Birth Research. A screenshot of the data repository resource as of January 2018. The landing page contains repository statistics, including the number of studies, participants, experimental samples, and study downloads. There are three sections including: 1) a table of the studies with corresponding links; 2) research highlights listing a selection of recent publications with links to the studies; and 3) a compilation of other relevant publicly-available datasets.

As of January 2018, the resource indexed 13 studies (Data Citations 1, 2, 3, 4, 5, 6, 7, 8, 9, 10, 11, 12, 13), with individual level molecular measurements on nearly 8,000 samples from more than 350 patients, capturing data from 6 measurement modalities (Fig. 2). The resource is further divided into three sections: 1) “curated datasets”; 2) “research highlights”; and 3) “other relevant publicly-available resources relevant to PTB research”. The resource is updated at least on a quarterly basis as new studies get added.

The studies referenced by the database (Table 1) cover a diverse set of modalities including microbiome, CyTOF, RNA-Seq, cell-free DNA and RNA sequencing, and genotyping. The protocols for data generation are included in each study (Fig. 1), as are subject demographics and relevant clinical variables as well as processed ‘omics’ datasets. When possible, raw data stored in other public repositories such as SRA, dbGaP and GEO are linked. Publications arising from the studies are referenced on individual study pages, and the most recent papers are also listed on the “research highlights” section of the resource.

Publicly-available data are displayed in a table format, which currently lists 15 additional studies from GEO, dbGaP, SRA, and ImmPort (Table 2). Various types of transcriptomic, microbiome, and genomic data across a variety of relevant reproductive tissues are included in the database with molecular profiles for more than 25,000 samples. The studies are linked from the resource page to their respective study detail pages in their respective repositories.

## Technical Validation

**Figure 3:**
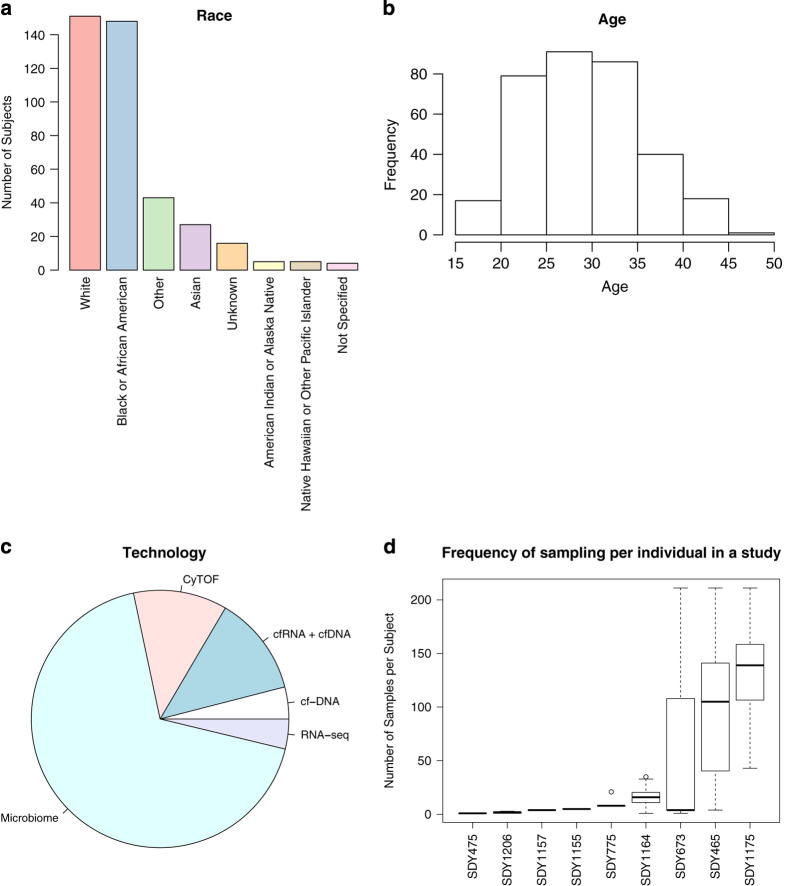
Overall Database Statistics on Individual Measurements (9/13 studies). (**a**) Race distribution across the cohort. (**b**) Age distribution across the cohort. (**c**) Percentage of samples profiled by each technology from the individual measurements. (**d**) Frequency of sampling per individual in each study.

In order to technically validate the resource, we computed some overall statistics on the repository (Fig. 3) focusing on the studies where we had individual level data (as opposed to aggregated summary statistics). As of January 2018, the database cohort consisted primarily of samples from White and African-American individuals with smaller proportions of samples from Asian and other racial and ethnic groups (Fig. 3a). The age of participants that we have individual level data on ranged from 16 to 46 years of age with a mean of 29.7±6.1 years (Fig. 3b). The majority of individuals (67.9%) in the resource have been profiled with microbiome 16s technology (Fig. 3c). There is a significant proportion of cell-free DNA and cell-free RNA measurements, as well as CyTOF (12.5 and 11.8% respectively). While the majority of the studies are cross-sectional and have very few samples per individual, there are several longitudinal studies (SDY465, SDY763 and SDY1175) with extensive sampling frequency (Fig. 3d).

We have also been tracking downloads through ImmPort for individual studies as well as bulk downloads as part of the ALLSTUDIES package, which contains the entirety of publicly-available ImmPort studies (Table 3). Individual study downloads counts range from 0 to 61 downloads with an average of 20 downloads per study in addition to a total of 666 downloads of the whole repository as of January 2018.

## Usage Notes

The overarching goal of our data-sharing effort is to enable new scientific discoveries from the rich molecular resources that have been funded by the MOD to advance the research in PTB. By making all the data publicly available, we aim to engage the larger research community in tackling this important problem. We are also working on incorporating other types of data into this repository including activity monitoring, sociobehavioral data, imaging and others. In addition to the traditional ‘omics’, investigators are studying non-coding regulatory regions, role of mitochondria, patient-specific glycans and sleep-wake cycles in the context of birth timing. We hope our efforts will entice industry leaders in the areas of machine learning and artificial intelligence to develop and apply computational methodologies to PTB research by leveraging the data that we aggregate. Our group has had tremendous success in mining publicly-available datasets to enable computational drug discoveries in the areas of autoimmunity and cancer^39–45^. More recently, we have carried out an integrative ancestry-specific GWAS analysis in PTB by integrating internal- and publicly-available data and identified several variants associated with early PTB^38^ as well as a transcriptomics meta-analysis of maternal and fetal signals elucidating the role of the immune system in parturition timing^46^. We will continue similar efforts in the area of PTB, and we hope to inspire others to follow suit. We hope this work enabling intersection of data layers related in the context of preterm birth will lead to discovery of novel diagnostic biomarkers and ultimately aid in formulating more effective interventional strategies for the management and prevention of PTB.

## Additional information

**How to cite this article**: Sirota, M. *et al.* Enabling precision medicine in neonatology, an integrated repository for preterm birth research. Sci. Data. 5:180219 doi: 10.1038/sdata.2018.219 (2018).

**Publisher’s note**: Springer Nature remains neutral with regard to jurisdictional claims in published maps and institutional affiliations.

## Supplementary Material



## Figures and Tables

**Table 1:** MOD-funded studies in the MOD database for preterm birth research as of January 2018.

**ID**	**Type of Data**	**Patients**	**Samples**	**Principal Investigator**	**Center**	**Unprocessed Data**	**Processed Data**	**PubMed ID**
***SDY465 (Data Citation 1)***	Microbiome 16S	***47***	***4,122***	David Relman	Stanford University	Raw reads Link to SRA	.BIOM format	26283357^26^
***SDY475 (Data Citation 2***	CYTOF	***23***	***95***	Martin Angst	Stanford University	3,000,000 cells per sample	Cell Clusters	26190063^27^
***SDY775 (Data Citation 3)***	Microbiome 16S	***7***	***69***	Samuel Parry and Frederic Bushman	University of Pennsylvania	Raw reads	.BIOM format	27338728^28^
***SDY1155 (Data Citation 4)***	RNA-Seq	***15***	***75***	Catalin Buhimschi	Ohio Collaborative	Sequencing	Counts matrix	27452435^29^
***SDY1164 (Data Citation 6)***	Microbiome 16s	***136***	***2,177***	David Relman	Stanford University	Raw reads	.BIOM	28847941^30^
***SDY1157 (Data Citation 7)***	*CYTOF*	***28***	***112***	Nima Aghaeepour and Brice Gaudilliere	Stanford University	3,000,000 cells per sample	Cell clusters	28864494^31^
***SDY673 (Data Citation d8)***	Cell Free DNA and RNA Seq	***50***	***188***	Stephen Quake	Stanford University	~20,000,000 reads per sample	Counts matrix	28904056^32^
***SDY1175 (Data Citation 8)***	Cell Free DNA Seq	***16***	***58***	Stephen Quake	Stanford University	~20,000,000 reads per sample	Abundance matrix	28830999^33^
SDY776* (Data Citation 9)	Genotyping	***1,705***	***1,705***	Louis Muglia	Ohio Collaborative	Genotypes Link to dbGaP	Association test results	26284790^34,35^
SDY1173* (Data Citation 10)	Genotyping	***~40k***	***~40k***	Louis Muglia	Ohio Collaborative	Genotypes Link to dbGaP	Association test results	28877031^35^
SDY1206* (Data Citation 11)	Microbiome	***77***	***149***	Molly Stout	Washington University in St. Luis	Raw reads link to Short Read Archive (SRA)	Abundance matrix	28549981^36^
SDY1215* (Data Citation 12)	Genotyping (Mitochondria)	***~15k***	***~15k***	Neal Sondheimer	University of Pennsylvanya in collab with Stanford	Genotypes Link to dbGaP	Association test results	29249523^37^
SDY1205* (Data Citation 13)	Genotyping	***~15k***	***~15k***	Marina Sirota and Atul Butte	Stanford in collab with Ohio Collaborative	Genotypes Link to dbGaP	Association test results	29317701^38^
*No individual level genomic data is available, only summary statistics and results.								

**Table 2:** Other publicly available studies relevant to preterm birth as of January 2018.

**Study ID**	**Database**	**Type**	**Number of Samples**	**Tissue**
GSE46510	GEO	Transcriptomics	154 samples	Maternal Blood
GSE59491	GEO	Transcriptomics	326 samples	Maternal Blood
GSE73685	GEO	Transcriptomics	183 samples	Amnion, Cord Blood, Decidua, Maternal Blood
phs001320.v1.p1	dbGaP	Transcriptomics	58 samples	Chorionic villus sampling
PRJNA242473	SRA	Microbiome	349 samples	Vaginal Microbiome
phs000735.v1.p1	dbGaP	Microbiome	48 samples	Placenta
phs000256.v3.p2	dbGaP	Microbiome	3,474 samples	Vaginal Microbiome
phs000332.v2.p2	dbGaP	Genetics	1,779 samples	Blood
phs000714.v1.p1	dbGaP	Genetics	2,928 samples	Blood
phs000353.v1.p1	dbGaP	Genetics	3,478 samples	Blood
phs000103.v1.p1	dbGaP	Genetics	2,000 mother-child pairs	Blood
phs001055.v1.p1	dbGaP	Genetics	2,397 samples	Blood
phs000276.v2.p1	dbGaP	Genetics	5,415 samples	Blood
SDY37	ImmPort	ELISA, Cytokines	55 samples	Maternal Blood
SDY36	ImmPort	Vaccine Response	300 samples	Maternal Blood
*No individual level genomic data is available, only linking out to the relevant resources.				

**Table 3:** Download statistics for each study as of January 2018.

**Study**	**First Data Release**	**Date of First Data Release**	**Number of downloads as of 01/22/2018**
**SDY465**	DR18	March 18, 2016	41
**SDY475**	DR16	December 14, 2015	54
**SDY673**	DR24	November 7, 2017	5
**SDY775**	DR20	January 31, 2017	27
**SDY776**	DR24	November 7, 2017	1
**SDY1155**	DR23	September 29, 2017	8
**SDY1157**	DR23	September 29, 2017	13
**SDY1164**	DR23	September 29, 2017	40
**SDY1173**	DR24	November 7, 2017	5
**SDY1175**	DR24	November 7, 2017	8
**SDY1205**	DR25	January 4, 2018	61
**SDY1206**	DR25	January 4, 2018	0
**SDY1215**	DR25	January 4, 2018	0
**Total**			**263**
**Bulk Download**	**666**		
