# Author Correction: Enabling precision medicine in neonatology, an integrated repository for preterm birth research

**DOI:** 10.1038/s41597-018-0004-3

**Published:** 2018-12-18

**Authors:** Marina Sirota, Cristel G. Thomas, Rebecca Liu, Maya Zuhl, Payal Banerjee, Ronald J. Wong, Cecele C. Quaintance, Rita Leite, Jessica Chubiz, Rebecca Anderson, Joanne Chappell, Mara Kim, William Grobman, Ge Zhang, Antonis Rokas, Louis J. Muglia, Carol Ober, Sarah K. England, George Macones, Deborah Driscoll, Samuel Parry, Gary M. Shaw, David K. Stevenson, Joe Leigh Simpson, Elizabeth Thomson, Atul J. Butte, March of Dimes Prematurity Research Centers, Deborah Driscoll, George Macones, Louis J Muglia, Carole Ober, David K. Stevenson

**Affiliations:** 1 Institute for Computational Health Sciences, University of California, San Francisco, CA 94158 USA; 2 Department of Pediatrics, University of California, San Francisco, CA 94158 USA; 3 Northrop Grumman Health Solutions, Rockville, MD 20850 USA; 4 March of Dimes, White Plains, NY 10605 USA; 5 Enterprise Science And Computing, Inc., Rockville, MD 20850 USA; 6 March of Dimes Prematurity Research Center at Stanford, Department of Pediatrics, Stanford University School of Medicine, Stanford, CA 94305 USA; 7 Perelman School of Medicine at the University of Pennsylvania, Philadelphia, PA 19104 USA; 8 Department of Obstetrics and Gynecology, Washington University in St Louis, St. Louis, MO 63110 USA; 9 Department of Human Genetics, University of Chicago, Chicago, IL 60637 USA; 10 Cincinnati Children’s Hospital Medical Center and University of Cincinnati College of Medicine, Cincinnati, OH 45267 USA; 11 Department of Biological Sciences, Vanderbilt University, Nashville, TN 37235 USA; 12 Department of Biomedical Informatics, Vanderbilt University Medical Center, Nashville, TN 37212 USA; 13 Department of Obstetrics and Gynecology, Feinberg School of Medicine, Northwestern University, Chicago, IL 60637 USA

Correction to: *Scientific Data* 10.1038/sdata.2018.219, published online 6 November 2018

The original version of the Data Descriptor contained errors in the author list and affiliations. Rita Leite’s first name was misspelled as “Rite” and affiliations 4 and 5 were incorrectly swapped. In addition, members of the March of Dimes Prematurity Research Center consortium were not listed in the agreed positions within the author list. These errors have now been corrected in the HTML and PDF versions.

